# Cumulative Lifespan Stress, Inflammation, and Racial Disparities in Mortality Between Black and White Adults

**DOI:** 10.1001/jamanetworkopen.2025.54701

**Published:** 2026-01-26

**Authors:** Isaiah D. Spears, Aaron J. Gorelik, Sara A. Norton, Michael J. Boudreaux, Megan W. Wolk, Jayne Siudzinski, Sarah E. Paul, Mary A. Cox, Cynthia E. Rogers, Thomas F. Oltmanns, Patrick L. Hill, Ryan Bogdan

**Affiliations:** 1 Department of Psychological and Brain Sciences, Washington University in St Louis, St Louis, Missouri; 2 Hogan Assessment Systems, Tulsa, Oklahoma; 3 Department of Psychiatry, Washington University School of Medicine, St Louis, Missouri

## Abstract

**Importance:**

In the US, Black individuals experience higher mortality risk than White individuals. Greater stress exposure and its biological sequelae are theorized to drive this heightened risk.

**Objective:**

To evaluate whether greater cumulative lifespan stress exposure and elevated inflammation are associated with increased mortality risk among Black compared with White US individuals.

**Design, Setting, and Participants:**

This longitudinal cohort study (St Louis Personality and Aging Network) recruited participants from 2007 to 2011 from the St Louis, Missouri, metropolitan area. Participants were required to have a mailing address to permit longitudinal follow-up and sixth grade reading level to facilitate understanding of the consent form; active psychosis was excludsionary. Data were analyzed from April to September 2025.

**Exposure:**

A latent lifespan cumulative stress factor was derived by applying bifactor confirmatory analysis to assessments of childhood maltreatment, lifetime trauma exposure, research assistant–verified stressful life events, major experiences of discrimination, and indices of socioeconomic status (ie, highest level of self and parental education, and annual household income).

**Main Outcomes and Measures:**

C-reactive protein (CRP) and interleukin-6 (IL-6) were measured from serum collected from 2014 to 2019 following all indices included in the cumulative stress factor. A CRP–IL-6 composite was formed. Mortality and cause of death were obtained from the Centers for Disease Control and Prevention National Death Index queried in December 2023. Linear regression and accelerated failure time models estimated mediational effects (ie, direct and indirect associations).

**Results:**

Among 1554 participants (505 Black [32.5%]; 1049 White [67.5%]; 853 [54.9%] female; mean [SD] age at baseline, 58.1 [2.9] years), Black individuals had shorter survival times than White individuals (time ratio, 0.937 [95% CI, 0.918 to 0.957]). Cumulative lifetime stress and the CRP–IL-6 composite, which were each higher among Black compared with White individuals, partially mediated the associations between race and mortality, accounting for 49.3% of racial disparities in mortality (indirect associations: serial, *b* = −0.006 [95% CI, −0.008 to −0.044]; cumulative stress, *b* = −0.009 [95% CI, −0.017 to −0.002]; CRP–IL-6, *b* = −0.016 [95% CI, −0.025 to −0.009]).

**Conclusions and Relevance:**

In this cohort study of St Louis adults, heightened cumulative lifespan stress and elevated inflammation were associated with shorter survival among Black participants, suggesting these pathways may represent plausible mechanisms mediating racial disparities in mortality among Black and White US individuals. The findings underscore the need for policies that address structural racism, alongside treatments that reduce inflammation and limit stress exposure to reduce mortality disparities.

## Key Points

**Question:**

Are elevated cumulative stress across the lifespan and inflammation associated with racial disparities in mortality between Black and White populations?

**Findings:**

Within this longitudinal cohort study of 1554 individuals, greater cumulative stress across the lifespan and higher inflammation partially mediated elevated mortality among Black participants.

**Meaning:**

These findings suggest that heightened cumulative stress and elevated inflammation are plausible mechanisms through which mortality disparities arise between Black and White individuals; there is a continued need for preventions, interventions, and policies that limit stress exposure and its potential impacts on health to reduce racial disparities in mortality.

## Teaser

This cohort study examines whether greater cumulative lifespan stress exposure and elevated inflammation are associated with increased mortality risk among Black compared with White individuals in the US.

## Introduction

There are substantial differences in mortality risk between Black and White individuals in the US; between 1999 and 2020, the Black US population experienced more than 1.63 million excess deaths than the White US population.^1^ This increased mortality risk first emerges during the perinatal period and adolescence but is most pronounced in late life, with chronic conditions (eg, heart disease) being the largest contributors.^1^ Elevated exposure to chronic stress resulting from systemic and explicit discrimination across generations and its biological sequelae, including inflammation, are elements of the weathering hypothesis and are believed to contribute to elevated mortality risk among Black individuals.^2,3^ Although data support stress and inflammation as contributors to excess disease and poor health outcomes among Black individuals,^4^ their putative role in mortality has yet to be investigated.^5,6,7^ Here, using data from participants in the ongoing longitudinal Saint Louis and Personality Aging Network (SPAN) Study, we tested whether cumulative stress across the lifespan (a latent factor derived from multiple self-report surveys) and inflammation in later life (measured by a C-reactive protein [CRP] and interleukin-6 [IL-6] composite [CRP–IL-6]) are associated with racial disparities in mortality among Black compared with White individuals. We hypothesized that higher levels of stress and inflammation would account for a substantive portion of increased mortality risk among Black individuals during later life in longitudinal mediation models.

## Methods

### Participants

The SPAN study recruited 1630 late middle-aged adults from the St Louis, Missouri, metropolitan area from 2007 to 2011 and observed them using in-person and mailed and/or online follow-up sessions for up to 17 years into older adulthood according to a research protocol approved by the Washington University Institutional Review Board (eMethods and eFigure 1 in Supplement 1). Written informed consent was obtained from all participants. This study was reported according to the Strengthening the Reporting of Observational Studies in Epidemiology (STROBE) reporting guidelines for cohort studies.

### Measures

#### Race

Participants reported their race (eMethods in Supplement 1). Only those who identified as Black or White and had not died before an inflammation assessment are included here.

#### Cumulative Stress

A latent lifespan cumulative stress factor was derived by applying bifactor confirmatory analysis to assessments of (1) childhood maltreatment (Childhood Trauma Questionnaire), (2) adult lifetime trauma exposure (Traumatic Life Events Questionnaire), (3) researcher-verified stressful life events (List of Threatening Experiences), (4) discrimination (Major Experiences of Discrimination), and (5) indices of socioeconomic status (ie, participant annual household income and highest level of education of participants and their parents) (eMethods in Supplement 1). Measures were collected until 2014 to 2016 (mean age at conclusion, 65.9 years; range, 60-73 years).

#### Inflammation

CRP and IL-6 were assayed from morning fasting serum samples collected between 2014 and 2019 (mean age, 66.7 years; range, 60-75 years). A CRP–IL-6 composite was formed by *z *scoring and averaging these moderately correlated (*r* = 0.494; *P* < .001) inflammatory markers (eTable 1 in Supplement 1).

#### Mortality

Mortality cause (eFigure 2 in Supplement 1) and exact date were obtained from the US Centers for Disease Control and Prevention National Death Index queried in December 2023. Time to event was coded as date of death minus date of birth (eMethods in Supplement 1).

### Statistical Analysis

CRP and IL-6 values were log-transformed, and all continuous data outliers were winsorized to 3 SD to minimize the influence of outliers prior to analyses. Linear regression and accelerated failure time models estimated whether cumulative stress and CRP–IL-6 mediate the association between race and mortality. Indirect associations (ie, serial: race, followed by stress, followed by CRP–IL-6, followed by mortality; independent stress: race, followed by stress, followed by mortality; independent inflammation: race followed by inflammation followed by mortality) were calculated via bootstrapping with 95% CIs using 10 000 iterations (eMethods in Supplement 1). Sex and age, reported by participants at study baseline, were included as covariates; post hoc analyses further included body mass index (BMI; calculated as weight in kilograms divided by height in meters squared) and medications as covariates on CRP–IL-6. The survival (version 3.5.5), mediation (version 4.5.0), survreg (version 3.8-3), and mets (version 1.3.5) packages in R statistical software version 4.3.1 (R Project for Statistical Computing) were used for analyses (eMethods in Supplement 1). Statistical significance was determined by 2-tailed *t* tests and χ^2^ tests, as well as 95% CIs, and was set at *P* < .05. Data were analyzed from April to September 2025.

## Results

There were a total of 1554 participants (505 Black individuals [32.5%]; 1049 White individuals [67.5%]; 853 women [54.9%]; mean [SD] age at baseline, 58.1 [2.9] years) (Table). Black participants experienced significantly shorter survival times (128 [25.3%] died; time ratio = 0.937 [95% CI, 0.918 to 0.957]; *P* < .001) (Figure, panels A and B), reported greater cumulative stress (*b* = 0.567 [95% CI, 0.493 to 0.641]) (Figure, panel C), and had higher CRP–IL-6 (*b* = 0.173 [95% CI, 0.110 to 0.238]) (Figure, panel D) than did White participants (125 White participants [11.9%] died) (Figure, panel E). Cumulative stress and CRP–IL-6 partially mediated racial disparities in mortality between Black and White individuals (total association [ie, race, stress, CRP–IL-6, mortality], *b* = −0.065 [95% CI, −0.086 to −0.044]; time ratio = 0.937 [95% CI, 0.918 to 0.957]; direct association [ie, race, mortality independent of stress and CRP–IL-6], *b* = −0.033 [95% CI, −0.055 to −0.01]; time ratio = 0.968 [95% CI, 0.947 to 0.990]). Significant serial (ie, race, stress, CRP–IL-6, mortality, *b* = −0.006 [95% CI, −0.008 to −0.044]), independent cumulative stress (ie, race, stress, mortality, *b* = −0.009 [95% CI, −0.017 to −0.002]), and inflammation (ie, race, CRP–IL-6, mortality, *b* = −0.016 [95% CI, −0.025 to −0.009]) indirect associations collectively accounted for 49.3% of the decreased survival time among Black compared with White individuals (Figure, panel E). The following alternative analytic strategies recapitulated these findings: (1) bivariate correlations (eTable 1 in Supplement 1), (2) single mediator (ie, stress or CRP–IL-6 only) models (eFigure 3 in Supplement 1), (3) CRP and IL-6 as independent markers (eFigure 4 in Supplement 1), (4) listwise deletion (eMethods and eFigure 5 in Supplement 1), and (5) including BMI and medications as covariates on CRP–IL-6 (eFigure 6 in Supplement 1). Additional results are shown in eTable 2 and eTable 3 in Supplement 1.

**Table.** Study Sample Demographic Information^a^

Characteristic	Participants, No. (%)		
	Black (n = 505 [32.5%])	White (n = 1049 [67.5%])	Total (N = 1554)
Age at baseline, mean (SD), y	57.9 (2.8)	58.1 (2.9)	58.1 (2.9)
Gender			
Female	282 (55.8)	571 (54.4)	853 (54.9)
Male	223 (44.1)	478 (45.6)	701 (45.1)
Annual household income, $			
<20 000	108 (21.9)	76 (7.5)	184 (11.3)
20 000-39 999	139 (28.1)	145 (14.2)	284 (17.4)
40 000-59 999	118 (23.9)	210 (20.6)	328 (20.1)
60 000-79 999	57 (11.5)	146 (14.3)	203 (12.5)
80 000-99 999	33 (6.7)	123 (12.1)	156 (9.6)
100 000-119 999	25 (5.1)	82 (8.1)	107 (6.6)
120 000-139 999	8 (1.6)	60 (5.9)	68 (4.2)
>140 000	6 (1.2)	176 (17.3)	182 (11.2)
Highest level of education			
Less than high school	18 (3.5)	9 (0.9)	27 (1.7)
High school or General Educational Development	113 (22.2)	102 (9.7)	220 (14.0)
Some college	126 (24.7)	158 (15.1)	284 (18.0)
Vocational training	52 (10.2)	34 (3.2)	86 (5.5)
2-y College degree	64 (12.6)	71 (6.6)	137 (8.7)
4-y College degree	83 (16.3)	314 (29.7)	397 (25.2)
Master’s degree	45 (8.8)	255 (24.1)	300 (19.0)
Professional or doctoral degree	9 (1.7)	114 (10.7)	123 (7.9)

^a^ There were no racial differences in age (*t* = 1.23; *P* > .22) or gender (χ^2^_1_ = 0.56; *P* = .45). Black participants had lower income (χ^2^_7_ = 192.93; *P* < .001) and education (χ^2^_7_ = 217.96; *P* < .001) than White participants.

**Figure.**
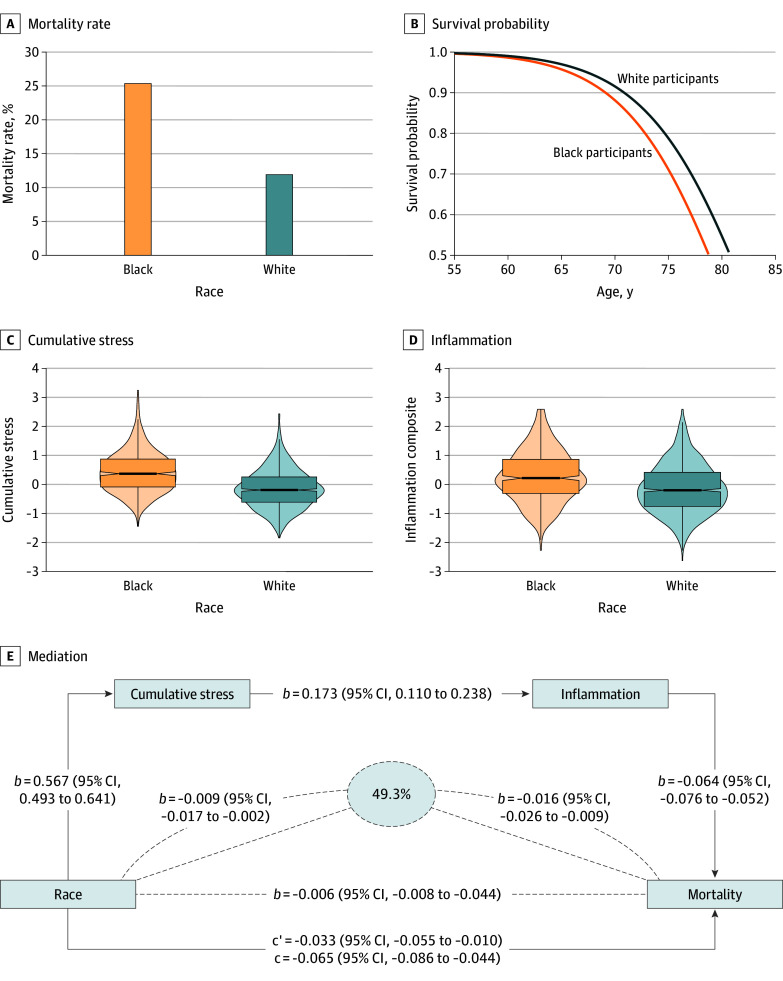
Results of Linear Regression and Accelerated Failure Time Models Compared with White participants, Black participants had higher mortality risk (A and B), cumulative stress (C), and inflammation (ie, C-reactive protein [CRP] and interleukin-6 [IL-6] composite) (D). Panel E shows that elevated cumulative lifespan stress and inflammation partially mediated the increased mortality risk among Black participants. Unstandardized path coefficients (*b*, *c*, and *c′*) and 95% CIs are presented. All regression parameters are reported as β coefficients (*b*); *c* denotes the β coefficient for the total association between race and mortality before accounting for relevant mediators (ie, cumulative stress and inflammation), and *c'* denotes the β coefficient for the association between race and mortality after accounting for mediators. Indirect associations are demarcated by dashed lines; the dashed line on the bottom reflects the serial indirect association; the upper dashed lines represent the independent cumulative stress and independent inflammation indirect associations. Race was coded as a dichotomous variable (Black = 1, White = 0). All variables were temporally ordered consistent with a longitudinal mediational model: cumulative stress was assessed prior to inflammation, and mortality was evaluated after both exposure and inflammatory biomarker data were collected.

## Discussion

Greater cumulative lifespan stress and inflammation accounted for a large proportion (ie, 49%) of the increased mortality risk among Black individuals compared with White individuals within our longitudinal cohort study in St Louis (1554 participants). These findings align with the weathering hypothesis, whereby cumulative exposure to structural and explicit discrimination increases stress and its biological consequences, contributing to premature health decline and mortality among Black individuals.^3^ Interventions and policies aimed at reducing cumulative stress, particularly those that address structural racism, alongside treatments that reduce inflammation,^6^ may attenuate mortality risk among US individuals broadly and narrow the health and mortality disparities between Black and White populations.

A substantive portion of mortality differences between Black and White individuals (>50%) were not accounted for by cumulative lifespan stress and inflammation. Alternative derivations of stress (eg, geospatial indices of neighborhood deprivation)^14^ and inflammation (eg, multiplex arrays), as well as moderators of their impact on health (eg, institutional health care trust and health care access),^7^ may account for additional variance. Nonetheless, the additional unexplained variance underscores the need to examine additional factors (eg, toxicant exposure, intergenerational epigenetic signatures, and social determinants of health)^8,15^ that have been independently linked to race, stress, and mortality-adjacent outcomes (eg, disease).^8,9^

### Limitations

This study has limitations that should be mentioned. Given substantial US regional variability in disparities in mortality between Black and White populations,^10^ it will be important to evaluate whether these findings generalize to other regions and evaluate locale-specific factors. Our cumulative lifespan stress factor reflects exposure to adverse experiences that may arise from structural and explicit discrimination. Future research will be needed to identify measurable factors that contribute to elevated stress exposure among Black individuals and to develop assessments of systemic discrimination exposure within individuals.^11^ In addition, consistent with frameworks that caution against interpreting racial health associations as a consequence of race,^12^ the indirect associations observed in our mediational model increase the plausibility that racial differences in exposure to cumulative lifespan stress and inflammation may drive racial disparities in mortality between Black and White individuals, while not establishing causality as residual confounding and epiphenomenal explanations cannot be ruled out.

## Conclusions

In this cohort study of Black and White individuals from the St Louis region, we found that cumulative lifespan stress and inflammation accounted for a large portion of the increased mortality risk among Black individuals. These findings underscore the continued need for preventions, interventions, and policies that limit stress exposure and its potential impacts on health to reduce mortality risk as well as mortality disparities between Black and White populations in the US.^13,14,15^

## Notes

Supplement 1. eMethods. eTable 1. Bivariate Correlations Among Variables of Interest eTable 2. Descriptive Statistics and Racial Group Differences on Health and Stress Variables eTable 3. 2010 American Community Survey (ACS) St. Louis City and County 1-Year Census Data eFigure 1. SPAN Study Timeline eFigure 2. Causes of Death Stratified by Race eFigure 3. Cumulative Stress and CRP/IL-6 Mediate Links Between Race and Mortality in Single Mediator Models eFigure 4. Cumulative Stress and CRP and IL-6 Mediate Links Between Race and Mortality: Models with CRP and IL-6 Individually Specified eFigure 5. Cumulative Stress and CRP and IL-6 Mediate Links Between Race and Mortality: Listwise Deletion Model eFigure 6. Cumulative Stress and CRP and IL-6 Mediate Links Between Race and Mortality: Accounting for BMI and Medication Associations with Inflammation eReferences.

Supplement 2. Data Sharing Statement
