# Supplementary material for: Cumulative Lifespan Stress, Inflammation, and Racial Disparities in Mortality Between Black and White Adults
Source: JAMA Netw Open. 2026 Jan 26;9(1):e2554701. doi: 10.1001/jamanetworkopen.2025.54701 (PMC12836128; doi:10.1001/jamanetworkopen.2025.54701)
Supplement: Supplement 1. — eMethods. eTable 1. Bivariate Correlations Among Variables of Interest eTable 2. Descriptive Statistics and Racial Group Differences on Health and Stress Variables eTable 3. 2010 American Community Survey (ACS) St. Louis City and County 1-Year Census Data eFigure 1. SPAN Study Timeline eFigure 2. Causes of Death Stratified by Race eFigure 3. Cumulative Stress and CRP/IL-6 Mediate Links Between Race and Mortality in Single Mediator Models eFigure 4. Cumulative Stress and CRP and IL-6 Mediate Links Between Race and Mortality: Models with CRP and IL-6 Individually Specified eFigure 5. Cumulative Stress and CRP and IL-6 Mediate Links Between Race and Mortality: Listwise Deletion Model eFigure 6. Cumulative Stress and CRP and IL-6 Mediate Links Between Race and Mortality: Accounting for BMI and Medication Associations with Inflammation eReferences. [file jamanetwopen-e2554701-s001.pdf]

Supplemental Online Content

Spears ID, Gorelik AJ, Norton SA, et al. Cumulative lifespan stress, inflammation, and racial disparities in mortality between Black and White adults. *JAMA Netw Open*. 2026;9(1):e2554701. doi:10.1001/jamanetworkopen.2025.54701

eMethods.
eTable 1. Bivariate Correlations Among Variables of Interest
eTable 2. Descriptive Statistics and Racial Group Differences on Health and Stress Variables
eTable 3. 2010 American Community Survey (ACS) St. Louis City and County 1-Year Census Data
eFigure 1. SPAN Study Timeline
eFigure 2. Causes of Death Stratified by Race
eFigure 3. Cumulative Stress and CRP/IL-6 Mediate Links Between Race and Mortality in Single Mediator Models
eFigure 4. Cumulative Stress and CRP and IL-6 Mediate Links Between Race and Mortality: Models with CRP and IL-6 Individually Specified
eFigure 5. Cumulative Stress and CRP and IL-6 Mediate Links Between Race and Mortality: Listwise Deletion Model
eFigure 6. Cumulative Stress and CRP and IL-6 Mediate Links Between Race and Mortality: Accounting for BMI and Medication Associations with Inflammation
eReferences.

This supplemental material has been provided by the authors to give readers additional information about their work.

© 2026 Spears ID et al. *JAMA Network Open*.

eMethods

SPAN Sample. The St. Louis Personality and Aging Network (SPAN) Study[1], which began in 2007, assesses biological, psychological, and social factors associated with health in later life among 1,630 late middle-aged adults aged 55-64 years of age at baseline (55% female; 65% White; 33% Black; 2% other). All participants in the cohort self-reported their race and ethnicity using the following survey categories: White/Caucasian; Black/African American; East Asian-Pacific Islander; South Asian; Middle Eastern; Native American; Biracial (write-in); and Other (write-in). Hispanic/Latino ethnicity was assessed separately with a "Yes/No" item followed by a write-in prompt for family background. The sample was recruited using standard epidemiological procedures, in which recruitment was aimed to be representative of the ethnic and socioeconomic composition of the late middle-aged St. Louis community at the time (46.9% male, 53.1.% female; 71.1% White, 19.3% Black, 9.6% not White or Black).[2] Due to low enrollment of Black males at the study origins, (31% of Black sample in 2007), intentional recruitment of Black males was used to ensure proper representation of the St. Louis community, which was successful (43% of Black sample; see Spence et al., 2011 and Oltmanns et al., 2011 for a detailed account of SPAN recruitment).[3,4] Inclusion criteria included a lack of active psychosis, ability to speak and read English (to understand consent and study materials), and ownership of an address and phone number (to allow future follow-ups). From 2007 to present day, SPAN participants completed regular in-person assessments (IPA) approximately every 3-4 years since baseline and remote assessments (RA) annually (**eFigure 1**) with additional subsequent assessments. All participants provided consent to participate in the SPAN study, which gained approval from the Institutional Review Board at Washington University in St. Louis (IRB#201102523). All procedures and reporting practices follow the STROBE (Strengthening the Reporting of Observational Studies in Epidemiology) guideline for cohort studies.

Inflammation. Fasting blood samples were collected in red top serum tubes between 7:30am and 10:00am via peripheral venipuncture during IPA2 and IPA3 (2014-2019; **eFigure 1**). Participants who reported any acute illness or injury were rescheduled. Immediately following collection, tubes were kept upright for 30-60 minutes to allow for clot formation, then centrifuged at 1300 rpm for 20 minutes. Samples were then immediately aliquoted and stored at -80 °C until the day of assay.

CRP and IL-6 were assayed from serum in duplicate using the commercially available enzyme-linked immunosorbent assay kits (DRG EIA-3954 High Sensitivity C-Reactive Protein ELISA, DRG International Inc., USA; sensitivity = 0.10 mg/L; Quantikine HS Human IL-6, R&D Systems, Minneapolis, MN, USA). Intra- (4%) and inter- (13%) assay coefficients of variation were acceptable. Data were log-transformed to address skew and allow for the inclusion of outliers prior to analysis. If a participant did not have CRP/IL-6 data available from IPA2, then CRP/IL-6 data from IPA3 was substituted in its place (n = 100). CRP and IL-6 were standardized (z-scored) within timepoint and averaged to form a composite inflammation index. Within person change was not examined given evidence of stability over time (rs=0.547-0.602).

© 2026 Spears ID et al. JAMA Network Open.

Cumulative Stress. We previously[5] derived a latent lifespan cumulative stress factor from participants in the SPAN cohort by applying bifactor confirmatory analysis using maximum likelihood estimation to assessments of 1) childhood maltreatment (Childhood Trauma Questionnaire; CTQ),[6] 2) adult lifetime trauma exposure (Traumatic Life Events Questionnaire; TLEQ),[7] 3) stressful life events (List of Threatening Experiences; LTE); each event was verified by research assistants through phone calls and endorsement subsequently modified as needed in consultation with participants,[8] 4) discrimination (Major Experiences of Discrimination; MED),[9] and 5) indices of socioeconomic status (i.e., highest level of education and annual household income). All variables were loaded onto a general factor, with the various indices of stress (CTQ, TLEQ, LTE, MED, SES) serving as indicators. Given the focus of the current study, we used only the general factor of cumulative stress in our analysis, as this represents the accumulation of chronic stress experienced across the lifespan. The model showed adequate fit (RMSEA = 0.022; CFI = 0.918; TLI = 0.908), and factor loadings on the cumulative stress factor ranged from -0.45 to 0.66 (average absolute loading of |0.34|). The general cumulative lifespan stress factor includes data from childhood to later life collected prior to the collection of inflammation and mortality data (**eFigure 1**). The measure, collection time, and assessment time of each individual measure contributing to this cumulative lifespan stress factor is described further below.

Childhood Maltreatment. Childhood maltreatment was assessed between 2014-2016 (**eFigure 1**) using the 28-item Childhood Trauma Questionnaire (CTQ),[6] which retrospectively measured indices of emotional abuse, emotional neglect, physical abuse, physical neglect, and sexual abuse before 17 years of age. The CTQ demonstrated good reliability and validity, with alphas ranging from 0.93 (sexual abuse) to 0.73 (physical neglect; Mdn. = 0.87) in the current sample. Scores for each of the five subscales of the CTQ were included in the factor analysis.

Childhood and Adult Socioeconomic Status (SES). Participants reported their annual household income, highest level of education and the highest level of education obtained by parents in 2007-2011 (baseline; **eFigure 1**). Annual household income was selected from a list of eight categories ranging from "Under $20,000" to "$140,000" or more." Self, maternal, and paternal education were selected from a list of nine categories ranging from "less than high school" to "professional degree (MD, JD, PhD)." Each (household income, participant education, maternal education, paternal education) were included as a measured variable in the cumulative stress factor analysis.

Adult Traumatic Life Events. Lifetime exposure to traumatic life events in adulthood were measured during 2010-2014 (IPA1; **eFigure 1**) using the 23-item Traumatic Life Events Questionnaire (TLEQ)[7]. Participants responded (yes/no) if they were exposed to traumatic events (e.g., physical assault). Questions pertaining to events in childhood were removed to prevent overlap with the CTQ. Each item was included as a measured variable in the factor analysis.

Lifetime Discrimination. The Major Experiences of Discrimination scale (MED),[9] which was administered between 2014-2016 (IPA2; **eFigure 1**), was used to assess lifetime experiences of discrimination across education, housing, employment, services, police, and healthcare. Participants indicated whether they had experienced discrimination in each domain of life for unfair reasons (e.g., race, gender, age) at any point in their lifetime. Each event was included as a measured variable in the cumulative stress factor analysis.

Stressful Life Events During Mid-Late Life. Stressful events (e.g., major transitions, financial stressors) occurring between 2008-2016 (RA1-RA4, IPA2-3; **eFigure 1**) that occurred between each assessment were measured using a 15-item checklist that combined the 12-item List of Threatening Experiences (LTE)[8] and three items added specifically for the current study (e.g., changes in family responsibilities). The number of life events endorsed were summed at each time point, with each sum included as a measured variable in the factor analysis.

© 2026 Spears ID et al. JAMA Network Open.

Mortality. Cause and the exact date of death were obtained from the National Death Index (NDI), a database maintained by the Centers for Disease Control and Prevention (CDC), on December 30th, 2023 (mortality assessment period: 2014-2023; **eFigure 1**). SPAN participants were linked to national death certificate data using identifying information (e.g., name, date of birth, social security number) to ensure accurate measurement of mortality outcomes. Mortality was coded in two ways: 1) a dichotomous variable indicating survival status (0 = alive, 1 = deceased) and 2) a continuous variable representing age at death, with participants who were still alive at the time of NDI data collection censored at their age on the NDI query date. All participants included were alive at the start of the inflammation assessment period, ensuring they had the opportunity to be assessed. Participants who died before the inflammation assessment period were excluded (n=23), leaving a total of 253 participants who died (128 Black, 125 White) during the mortality assessment period (i.e., November 1st 2014-December 30th, 2023).

Analyses. Linear regression and accelerated failure time (AFT) models were used to examine whether cumulative stress and CRP/IL-6 mediate the association between race and mortality. Mediation effects are reported as follows: 1) the *total association* represents the overall association between race and survival time, 2) the *direct association* is the association between race and survival time that is independent of stress and inflammation mediators, 3) the *serial indirect association* represents the sequential pathway (i.e., race → cumulative stress → inflammation → mortality), and 4) *independent indirect associations* reflect each mediator's independent contribution (i.e., race → cumulative stress → mortality; race → inflammation → mortality). Survival time was calculated as the difference between self-reported date of birth and the exact date of death recorded in the NDI. For participants who were alive on December 30th, 2023, data were coded indicating that the event had not occurred.

All analyses were conducted using R (version 4.4.1). Primary survival and mediation models were implemented using survival (version 3.5.5)[10], mediation (version 4.5.0)[11], survminer (version 0.5.0)[12], and mets (version 1.3.5)[13,14] packages. Multiple imputation of missing data was performed using predictive mean matching through the mice package (version 3.16.0)[15]. Bootstrapped serial mediation estimates were computed across 10,000 iterations per imputed dataset.

Supplementary Results. In order to evaluate robustness of findings, several alternative analytics strategies were conducted. Bivariate correlations (**eTable 1**) confirmed expected associations among race, cumulative stress, inflammation, and mortality. Single-mediator models (**eFigure 3**) indicated that both cumulative stress and inflammation independently mediated racial disparities in survival time. When CRP and IL-6 were modeled as separate inflammatory markers (**eFigure 4**), both exhibited comparable indirect effects, suggesting that the composite captured a shared inflammatory pathway. Analyses using listwise deletion (**eFigure 5**) replicated all key effects. Finally, given evidence that adipose tissue has immune properties that promote systemic inflammation,[16] we included a final analytic model wherein Body Mass Index (BMI) was included as a covariate on inflammation to evaluate whether the link between cumulative lifespan stress and inflammation was independent of BMI variability. Notably, while the BMI construct is limited by heterogeneity (e.g., potentially arising from excess adipose tissue, muscle mass, and/or different body types), BMI is highly correlated with direct measures of adipose tissue (e.g., bioimpedence, x-ray absorpiometry, and MRI, which were not available in our study) among individuals (n>500,000) in mid-later life.[17,18] This model (**eFigure 6**) did not meaningfully alter any total, direct, or indirect associations, supporting the robustness of primary findings.

© 2026 Spears ID et al. JAMA Network Open.

eTable 1. Bivariate Correlations Among Variables of Interest

Variable	1	2	3	4	5	6	7	8	9
1.Race	1	0.364**	0.206**	0.175**	0.204**	-0.033	-0.150**	0.020	0.176**
2. Cumulative Stress	0.364**	1	0.160**	0.168**	0.189**	-0.042	-0.121**	0.168**	0.136**
3. IL-6	0.206**	0.160**	1	0.494**	0.864**	-0.003	-0.248**	-0.025	0.333**
4. CRP	0.175**	0.168**	0.494**	1	0.864**	0.017	-0.156**	0.11**	0.22**
5. Inflammation Composite	0.204**	0.189**	0.864**	0.864**	1	0.004	-0.234**	0.05	0.319**
6. Age (Baseline)	-0.033	-0.042	-0.003	0.017	0.004	1	0.699**	0.028	0.052*
7. Age at Death/Censoring	-0.150**	-0.121**	-0.248**	-0.156**	-0.234**	0.699**	1	0.040	-0.544**
8. Sex	0.020	0.168**	-0.025	0.11**	0.05	0.028	0.04	1	-0.043
9. Mortality Status	0.176**	0.136**	0.333**	0.22**	0.319**	0.052*	-0.544**	-0.043	1

Note. Values are Pearson correlation coefficients calculated across five multiply imputed datasets. Sex is coded as 0 = Male and 1 = Female. Race is coded as 0 = White and 1 = Black. ** = correlation is significant at the 0.001 level (2-tailed). * = correlation is significant at the 0.05 level (2-tailed).

© 2026 Spears ID et al. JAMA Network Open.

eTable 2. Descriptive Statistics and Racial Group Differences on Health and Stress Variables

Measure		Black (M, SD)	White (M, SD)	Test Statistic (t / χ2)	Effect Size (d / φ)	Total (M, SD)
**Inflammation**	CRP	0.5 (0.5)	0.38 (0.5)	t = -4.39**	0.38	0.43 (0.5)
	IL-6	0.4 (0.3)	0.3(0.3)	t = -6.6**	0.4	0.31 (0.32)
**Childhood Maltreatment (CTQ)**	Physical Abuse	8.1 (3.4)	6.4 (2.5)	t = 8.57**	0.57	6.9 (2.9)
	Emotional Abuse	7.9 (4.0)	7.8 (4.0)	t = 0.56	0.03	7.8 (4.0)
	Sexual Abuse	6.7 (4.1)	5.8 (2.6)	t = 4.13**	0.26	6.1 (3.1)
	Physical Neglect	6.9 (2.8)	6.0 (2.1)	t = 5.01**	0.36	6.3 (2.4)
	Emotional Neglect	8.7 (4.5)	8.9 (4.3)	t = -0.63	-0.05	8.8 (4.4)
**Ongoing Stressful Events (LTE)**	Number of Events	2.8 (2.2)	2.1 (1.9)	t = 5.96**	0.34	2.3 (2.0)
**Adult Trauma Exposure (TLEQ)**	Number of Events	4.0 (2.6)	3.1 (2.1)	t = 5.97**	0.38	3.4 (2.3)
**Experiences of Discrimination (MED)**[a]	Education	51 (18.0%)	56 (7.6%)	χ2 = 24.0**	0.15	111 (10.6)
	Purchasing a house	41 (14.5%)	13 (1.8%)	χ2 = 66.2**	0.25	59 (5.6)
	Workplace	115 (41.1%)	179 (24.2%)	χ2 = 28.2**	0.17	306 (29.2)
	Denied medical service	23 (8.1%)	12 (1.6%)	χ2 = 26.3**	0.13	39 (3.7)
	Denied basic services	57 (20.4%)	83 (11.3%)	χ2 = 14.2**	0.12	144 (13.8)
	Police harassment	90 (31.9%)	57 (7.7%)	χ2 = 97.4**	0.31	159 (15.1)

Note. Descriptive statistics and racial group comparisons on individual health and stress variables are presented. For continuous variables, means (M) and standard deviations (SD) are presented for each group, with independent samples t-tests (*t*) used to

© 2026 Spears ID et al. JAMA Network Open.

assess group differences. [a]For categorical variables number and percentage of group sample (i.e., Black/White sample) endorsing the item are shown, along with chi-square ($\chi 2$)tests used to assess group differences.

© 2026 Spears ID et al. JAMA Network Open.

eTable 3. 2010 American Community Survey (ACS) St. Louis City and County 1-Year Census Data

Variable		Black Total<br>n = 32,133 (19.3%)	White Total<br>n = 118,114 (71.4%)	Total Population<br>n = 166,027
Gender[A]	Male	8,846 (27.5%)	57,253 (48.4%)	76,953 (46.9%)
	Female	23,287 (72.5%)	60,861 (51.6%)	87,272 (53.1%)
Income[B]	<20,000	17,241 (29.4%)	15,063 (10.0%)	40,857 (18.2%)
	20,000–39,999	15,676 (26.7%)	21,608 (14.3%)	38, 622 (17.2%)
	40,000–59,999	9,531 (16.2%)	22,590 (15.0%)	33,125 (14.8%)
	60,000–74,999	5,204 (8.8%)	15,418 (10.2%)	21,325 (9.5%)
	75,000–99,999	4,450 (7.5%)	23,320 (15.4%)	28,881 (12.9%)
	100,000–124,999	2,795 (4.7%)	15,048 (9.9%)	18, 634 (8.3%)
	125,000–149,999	1,645 (2.8%)	12,008 (7.9%)	14,067 (6.3%)
	>150,000	2,076 (3.5%)	25,534 (16.9%)	28, 674 (12.8%)
Education[C]	Less than 9th grade	9,750 (4.1%)	17,808 (2.9%)	29,515 (3.6%)
	9th–12th grade	34,394 (14.6%)	29,970 (4.8%)	66,950 (8.1%)
	Regular high school diploma	61,872 (26.4%)	117,341 (19.1%)	214, 547 (25.9%)
	GED or alternative credential	10,993 (4.7%)	17,002 (2.7%)	214, 547 (*)
	Some college, no degree	62,110 (26.5%)	117,721 (19.2%)	185, 550 (22.4%)
	Associate’s degree	16,428 (7.0%)	43,260 (7.0%)	62, 926 (7.6%)
	Bachelor’s degree	24,666 (10.5%)	162,072 (26.4%)	129,959 (15.7%)
	Graduate degree	13,835 (5.9%)	108,127 (17.6%)	140,730 (17.0%)

Combined 2010 American Community Survey (ACS) 1-Year population totals, income, and educational attainment of St. Louis County and City residents are presented by race and gender. Age ranges reflect ACS data available for each variable. [A] = population totals based on age ranges 55-64. [B] = income distributions for ages 45-64. [C] = educational attainment for residents aged 25 or older in St. Louis County and City. * = this category overlaps with another group in the population dataset (i.e., regular high school diploma) and is not an additional independent count. Percentages represent the proportion of each category within racial group totals and within the overall population. For example, income percentages reflect the share of White, Black, and total residents falling into each income bracket; education percentages reflect the distribution of educational attainment within each racial group and overall.

© 2026 Spears ID et al. JAMA Network Open.

eFigure 1. SPAN Study Timeline.

A)

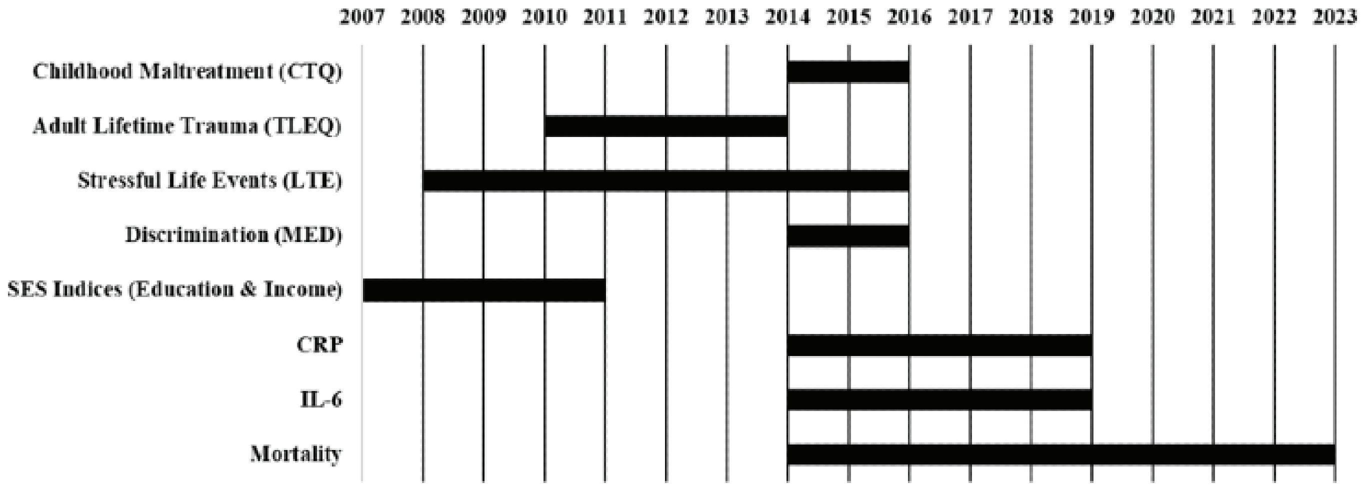

B)

Wave	Years	Location	Participant *N* (M_{age}, *Range)*
Baseline	08/07 - 4/11	Lab	1630 (58.1, 55-65)
RA1	2/08 - 10/11	Home	1435 (60.1, 55-65)
RA2	9/08 - 5/12	Home	1381 (60.7, 56-67)
RA3	3/09 - 11/12	Home	1326 (61.3, 56-67)
RA4	9/09 - 1/13	Home	1230 (62.1, 57-68)
IPA1[a]	3/10 - 11/12<br>6/14 - 12/14	Lab	1280 (62.5, 57-70)
IPA2	11/14 - 11/16	Lab	1072 (65.9, 60-73)
IPA3	11/16 - 11/19	Lab	1035 (68.1, 62-75)

SPAN Study collection timeline. **A.** Graphical representation of SPAN study timeline and measure collection. Bars represent the years during which each measure was collected. CTQ = Childhood Trauma Questionnaire, TLEQ = Traumatic Life Events Questionnaire, LTE = Lifetime Traumatic Events, MED = Major Experiences of Discrimination, CRP = C-reactive protein, IL-6 = Interleukin-6. Notably, temporal ordering was consistent with the longitudinal mediational model (i.e., race -> cumulative stress -> inflammation composite -> mortality); assessments of stress all preceded inflammation assessments, and the mortality period evaluated began after each participant provided a blood sample for inflammation analyses.

B) Table representing the duration of each data collection wave in SPAN. [a]The St. Louis Personality and Aging Network (SPAN) study experienced a gap in study funding between 11/2012 and 06/2014. As a result, data collection for IPA1 was extended. 1,005 participants completed IPA1 between 3/10 – 11/12 and 272 participants completed IPA1 between 06/14 and 12/14. RA = Remote Assessment; IPA = In-Person Assessment.

© 2026 Spears ID et al. JAMA Network Open.

eFigure 2. Causes of Death Stratified by Race.

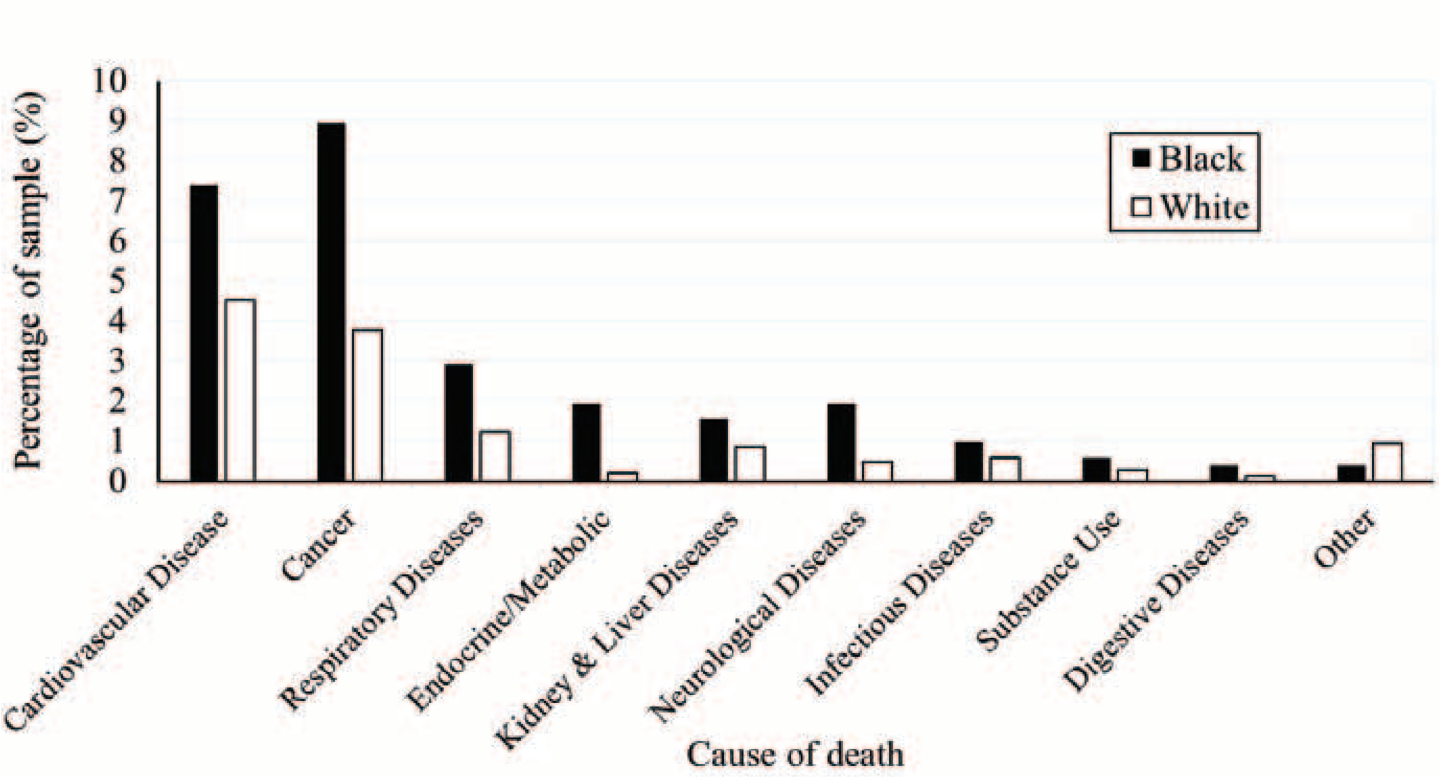

eFigure 2. Bars represent the percentage of individuals within each racial group (Black or White) who died from each cause of death. Overall, 25.34% of Black participants (128 of 505) and 11.91% of White participants (125 of 1049) died over the follow-up period. Compared with White participants, Black participants experienced higher proportions of death due to cardiovascular disease (7.38% vs 4.52%; $\chi^2(1) = 5.50$, $p < 0.05$), cancer (8.93% vs 3.77%; $\chi^2(1) = 17.95$, $p < 0.001$), respiratory disease (2.91% vs 1.22%; $\chi^2(1) = 5.67$, $p < 0.017$), endocrine or metabolic conditions (1.94% vs 0.19%; $\chi^2(1) = 14.12$, $p < 0.001$), and neurological diseases (1.94% vs 0.47%; $\chi^2(1) = 7.97$, $p < 0.001$). There was no significant difference in proportion of deaths due to kidney/liver diseases between Black and White participants (1.55% vs 0.85% ;$\chi^2(1) = 1.62$, $p = .203$), infectious diseases (0.97% vs 0.56%; $\chi^2(1) = 0.83$, $p = .364$), substance use (0.58% vs 0.28%; $\chi^2(1) = 0.82$, $p = .364$), digestive diseases (0.39% vs 0.09%; $\chi^2(1) = 1.58$, $p = .209$), and other (0.94% vs 0.39%; $\chi^2(1) = 1.41$, $p = .236$).

© 2026 Spears ID et al. JAMA Network Open.

eFigure 3. Cumulative Stress and CRP/IL-6 Mediate Links Between Race and Mortality in Single Mediator Models.

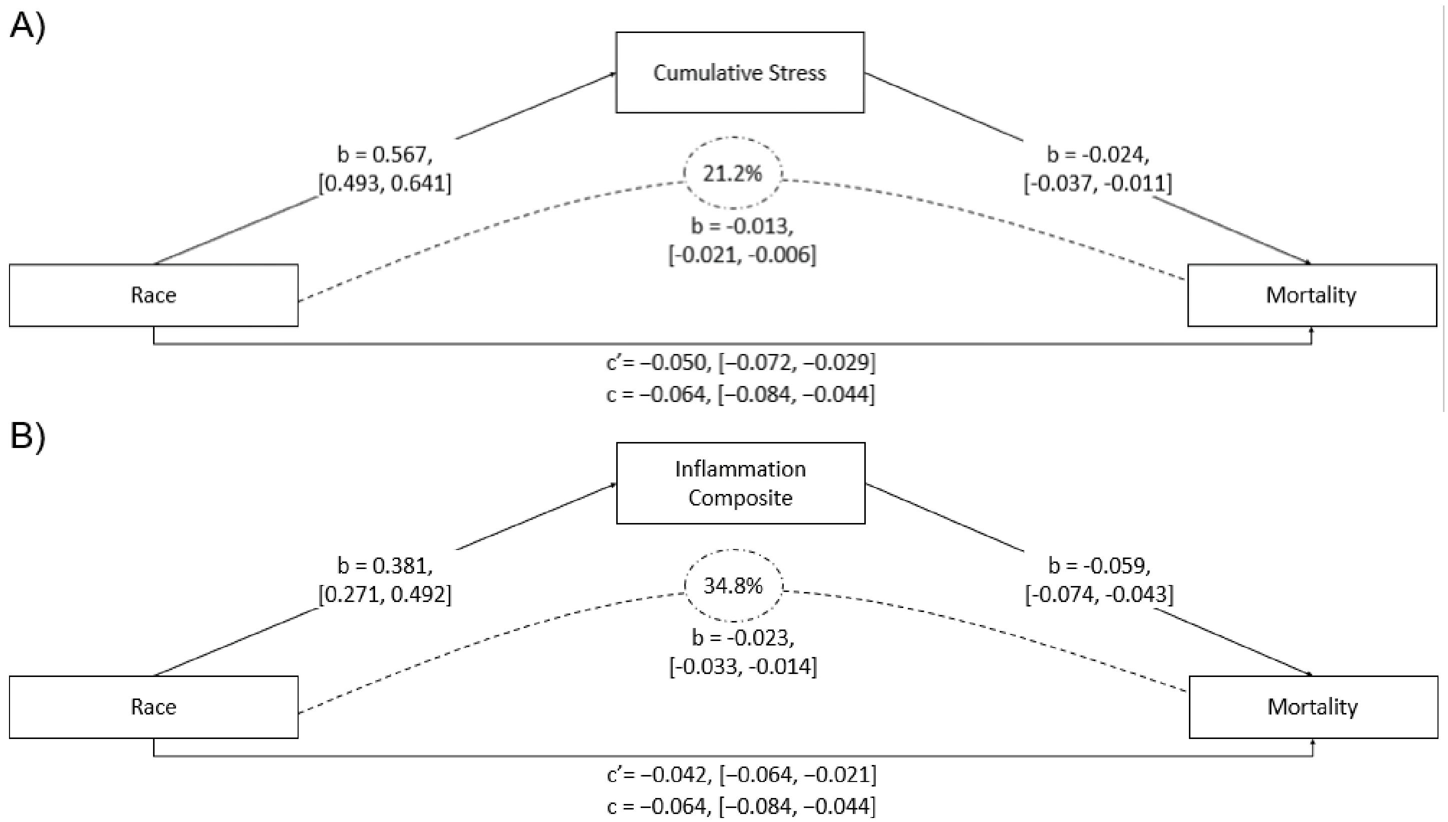

eFigure 3. Unstandardized path coefficients (*b*) and 95% confidence intervals are presented. Both cumulative stress (A) and inflammation (B) partially mediated the association between race and mortality in their respective single mediator models. Indirect effects are represented by the dashed line. The circled number represents the percent of the total effect that is accounted for by the mediator. *c* = total effect, *c'* = direct effect of Race on mortality independent of the mediator. All variables were temporally ordered consistent with a longitudinal mediational model: cumulative stress was assessed prior to inflammation, and mortality was evaluated after both exposure and inflammatory biomarker data were collected.

eFigure 4. Cumulative Stress and CRP and IL-6 Mediate Links Between Race and Mortality: Models with CRP and IL-6 Individually Specified.

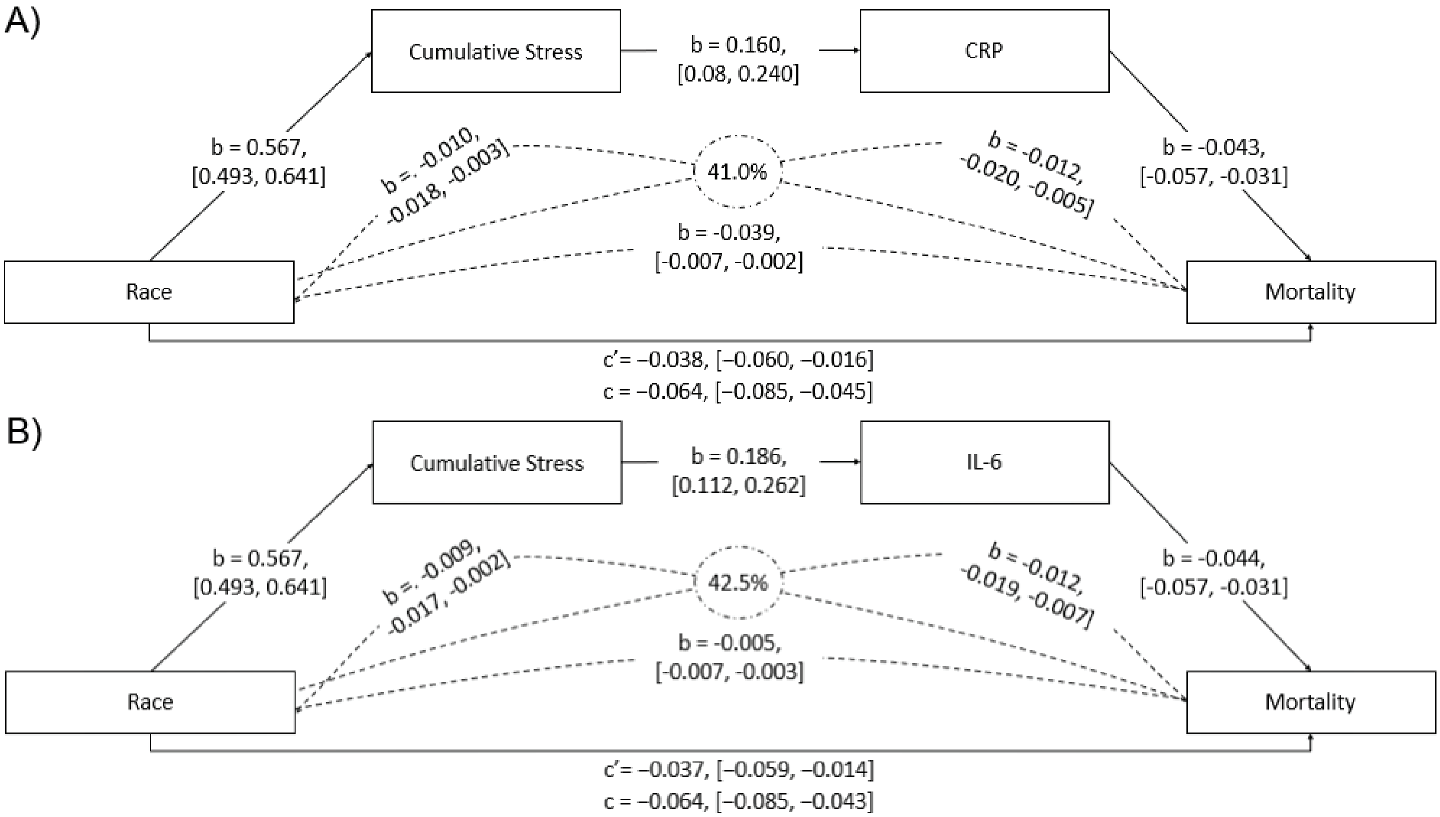

eFigure 4. AFT Serial mediation models in which CRP and IL6 are used in place of inflammation composite are shown. Unstandardized path coefficients (*b*) and 95% confidence intervals are presented. Cumulative stress and both CRP (A) and IL-6 (B) partially mediated the association between race and mortality in their respective serial mediation models. Indirect effects are represented by the dashed line. The circled number represents the percent of the total effect that is accounted for by the mediator. *c* = total effect, *c'* = direct effect of Race on mortality independent of the mediator. All variables were temporally ordered consistent with a longitudinal mediational model: cumulative stress was assessed prior to inflammation, and mortality was evaluated after both exposure and inflammatory biomarker data were collected.

© 2026 Spears ID et al. JAMA Network Open.

eFigure 5. Cumulative Stress and CRP and IL-6 Mediate Links Between Race and Mortality: Listwise Deletion Model

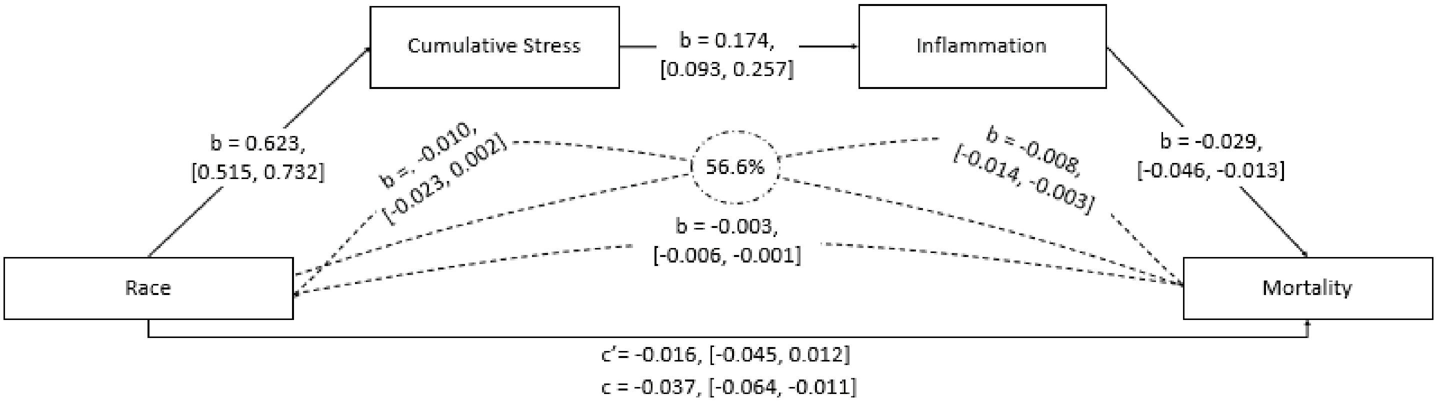

eFigure 5. This figure presents a serial mediation model examining whether cumulative stress and systemic inflammation (measured as a composite) jointly mediate the association between race and mortality using listwise deletion (i.e., including only participants with complete data on all variables). Unstandardized path coefficients (*b*) and 95% confidence intervals are presented. Indirect effects are represented by the dashed line. The circled number represents the percent of the total effect that is accounted for by the mediator. *c* = total effect, *c'* = direct effect of Race on mortality independent of the mediator. All variables were temporally ordered consistent with a longitudinal mediational model: cumulative stress was assessed prior to inflammation, and mortality was evaluated after both exposure and inflammatory biomarker data were collected.

© 2026 Spears ID et al. JAMA Network Open.

eFigure 6. Cumulative Stress and CRP and IL-6 Mediate Links Between Race and Mortality: Accounting for BMI and Medication Associations with Inflammation.

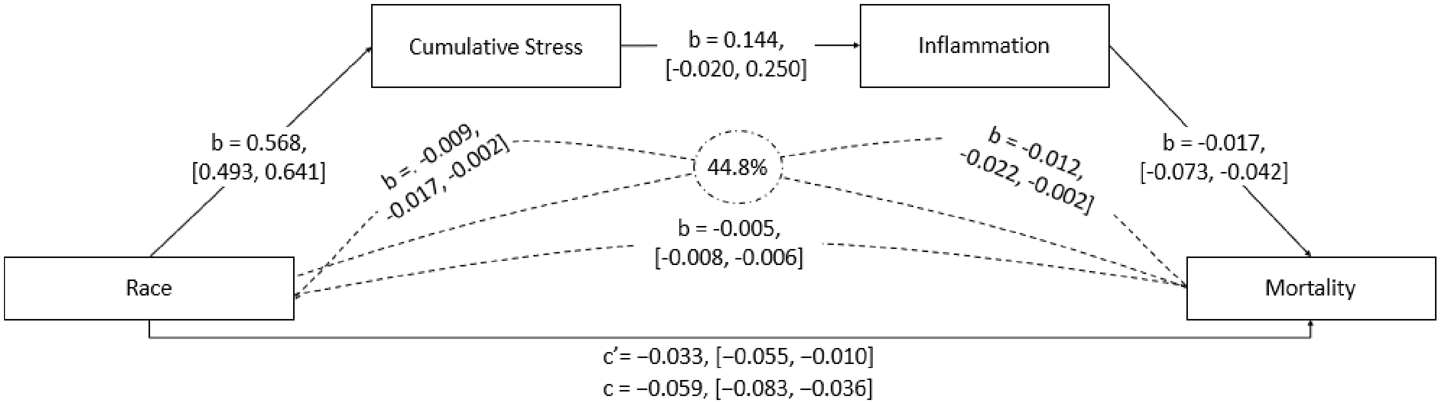

eFigure 6. This figure presents a serial mediation model testing whether cumulative stress and inflammation mediate the association between race and mortality risk. Body mass index (BMI) and mediation use were included as additional covariates on the inflammation pathway for this model, and age at baseline and gender were included as covariates throughout the model, consistent with all prior analyses. Unstandardized path coefficients (*b*) and 95% confidence intervals are presented. Indirect effects are represented by the dashed line. The circled number represents the percent of the total effect that is accounted for by the mediator. *c* = total effect, *c'* = direct effect of Race on mortality independent of the mediator. All variables were temporally ordered consistent with a longitudinal mediational model: cumulative stress was assessed prior to inflammation, and mortality was evaluated after both exposure and inflammatory biomarker data were collected.

© 2026 Spears ID et al. JAMA Network Open.

eReferences

1. Oltmanns, T. F., Rodrigues, M. M., Weinstein, Y. & Gleason, M. E. J. Prevalence of Personality Disorders at Midlife in a Community Sample: Disorders and Symptoms Reflected in Interview, Self, and Informant Reports. *J. Psychopathol. Behav. Assess.* **36**, 177–188 (2014).
2. U.S. Census Bureau. 2010 American Community Survey 1-Year Estimates. Age and Sex — St. Louis County, MO & St. Louis city, MO. (2010).
3. Spence, C. T. & Oltmanns, T. F. Recruitment of African American men: Overcoming challenges for an epidemiological study of personality and health. *Cultur. Divers. Ethnic Minor. Psychol.* **17**, 377–380 (2011).
4. Oltmanns, T. F. Personality, health, and social adjustment in later life. in *Mental health in public health: The next 100 years.* 151–179 (Oxford University Press, 2011).
5. McClendon, J., Chang, K., J. Boudreaux, M., Oltmanns, T. F. & Bogdan, R. Black-White racial health disparities in inflammation and physical health: Cumulative stress, social isolation, and health behaviors. *Psychoneuroendocrinology* **131**, 105251 (2021).
6. Bernstein, D. P., Fink, L., Handelsman, L. & Foote, J. Childhood Trauma Questionnaire. https://doi.org/10.1037/t02080-000 (2011).
7. Kubany, E. S. *et al.* Development and preliminary validation of a brief broad-spectrum measure of trauma exposure: The Traumatic Life Events Questionnaire. *Psychol. Assess.* **12**, 210–224 (2000).
8. Brugha, T., Bebbington, P., Tennant, C. & Hurry, J. The List of Threatening Experiences: a subset of 12 life event categories with considerable long-term contextual threat. *Psychol. Med.* **15**, 189–194 (1985).
9. Sternthal, M. J., Slopen, N. & Williams, D. R. RACIAL DISPARITIES IN HEALTH: How Much Does Stress Really Matter? *Bois Rev. Soc. Sci. Res. Race* **8**, 95–113 (2011).
10. Therneau, T. M. & Grambsch, P. M. *Modeling Survival Data: Extending the Cox Model*. (Springer, New York, 2000).
11. Tingley, D., Yamamoto, T., Hirose, K., Keele, L. & Imai, K. **mediation** : *R* Package for Causal Mediation Analysis. *J. Stat. Softw.* **59**, (2014).
12. Drawing Survival Curves using ggplot2. https://rpkgs.datanovia.com/survminer/index.html.

© 2026 Spears ID et al. JAMA Network Open.

13. Holst, K. K., Scheike, T. H. & Hjelmborg, J. B. The liability threshold model for censored twin data. *Comput. Stat. Data Anal.* **93**, 324–335 (2016).

14. Scheike, T. H., Holst, K. K. & Hjelmborg, J. B. Estimating heritability for cause specific mortality based on twin studies. *Lifetime Data Anal.* **20**, 210–233 (2014).

15. Buuren, S. V. & Groothuis-Oudshoorn, K. **mice** : Multivariate Imputation by Chained Equations in *R. J. Stat. Softw.* **45**, (2011).

16. Saltiel, A. R. & Olefsky, J. M. Inflammatory mechanisms linking obesity and metabolic disease. *J. Clin. Invest.* **127**, 1–4 (2017).

17. Jeong, S.-M., Lee, D. H., Rezende, L. F. M. & Giovannucci, E. L. Different correlation of body mass index with body fatness and obesity-related biomarker according to age, sex and race-ethnicity. *Sci. Rep.* **13**, 3472 (2023).

18. Wiebe, N. & Tonelli, M. Associations of body fat and inflammation with non-communicable chronic diseases and mortality: a prospective cohort study of the UK Biobank. *BMJ Open* **15**, e092962 (2025).

© 2026 Spears ID et al. JAMA Network Open.
