# Supplementary material for: Cumulative Lifespan Stress, Inflammation, and Racial Disparities in Mortality Between Black and White Adults
Source: JAMA Netw Open. 2026 Jan 26;9(1):e2554701. doi: 10.1001/jamanetworkopen.2025.54701 (PMC12836128; doi:10.1001/jamanetworkopen.2025.54701)
Supplement: Supplement 2. — Data Sharing Statement [file jamanetwopen-e2554701-s002.pdf]

Data Sharing Statement

Spears. Cumulative Lifespan Stress, Inflammation, and Racial Disparities in Mortality between Black and White Adults. *JAMA Netw Open*. Published January 20, 2026. doi:10.1001/jamanetworkopen.2025.54701

Data
Data available: Yes
Data types: Deidentified participant data
How to access data: Investigators interested in using data from the Saint Louis Personality and Aging Network (SPAN) Study may submit a data access request to Drs. Ryan Bogdan and Patrick Hill (rbogdan@wustl.edu, patrick.hill@wustl.edu) to obtain a Data Use Certificate. Analytic code is available here:https://github.com/WashU-BG.
When available: With publication

Supporting Documents
Document types: None

Additional Information
Who can access the data: Researchers whose proposed use of these data are approved.
Types of analyses: For an approved specified purpose.
Mechanisms of data availability: Investigators interested in using data from the Saint Louis Personality and Aging Network (SPAN) Study may submit a data access request to Drs. Ryan Bogdan and Patrick Hill (rbogdan@wustl.edu, patrick.hill@wustl.edu) to obtain a Data Use Certificate. Analytic code is available here:https://github.com/WashU-BG. IS and RB had full access to all data in the study and take responsibility for the integrity of the data and accuracy of the data analysis.
